# Thermal and Quasi-Static Mechanical Characterization of Polyamide 6-Graphene Nanoplatelets Composites

**DOI:** 10.3390/nano11061454

**Published:** 2021-05-31

**Authors:** Pietro Russo, Francesca Cimino, Antonio Tufano, Francesco Fabbrocino

**Affiliations:** 1 Institute for Polymers, Composites and Biomaterials, National Research Council, Via Campi Flegrei 34, 80078 Pozzuoli, Italy; francesca.cimino@ipcb.cnr.it; 2 Department of Engineering, Telematic University Pegaso, Centro Direzionale Napoli Isola F2, Pegaso Tower, 80143 Napoli, Italy; antonio.tufano@unipegaso.it (A.T.); francesco.fabbrocino@unipegaso.it (F.F.)

**Keywords:** polyamide 6, graphene nanoplatelets, thermal properties, mechanical properties

## Abstract

The growing demand for lightweight and multifunctional products in numerous industrial fields has recently fuelled a growing interest in the development of materials based on polymer matrices including graphene-like particles, intrinsically characterized by outstanding mechanical, thermal, and electrical properties. Specifically, with regard to one of the main mass sectors, which is the automotive, there has been a significant increase in the use of reinforced polyamides for underhood applications and fuel systems thanks to their thermal and chemical resistance. In this frame, polyamide 6 (PA6) composites filled with graphene nanoplatelets (GNPs) were obtained by melt-compounding and compared in terms of thermal and mechanical properties with the neat matrix processed under the same condition. The results of the experimental tests have shown that the formulations studied so far offer slight improvements in terms of thermal stability but much more appreciable benefits regarding both tensile and flexural parameters with respect to the reference material. Among these effects, the influence of the filler content on the strength parameter is noteworthy. However, the predictable worsening of the graphene sheet dispersion for GNPs contents greater than 3%, as witnessed by scanning electron images of the tensile fractured sections of specimens, affected the ultimate performance of the more concentrated formulation.

## 1. Introduction

In the last decades, the addition of nanoscale fillers to enhance specific properties of polymers has triggered intensive research activities in science and engineering. Nanoscale fillers such as nanoclays, carbon nanotubes, and graphene appear ideal to develop new multi-functional materials [1,2], but the more their potential is transmitted to the products, the better they are dispersed in the host polymeric matrix and the greater the interactions between the filler and the latter. These conditions, achievable by solution mixing and in situ polymerization methods, are usually difficult to obtain by melt compounding, which, on the other hand, is the simplest, cheapest, and most scalable technology on industrial levels [3]. In this case, the prevalence of filler–filler interactions over filler–matrix ones and high melt viscosity represent technological challenges to be faced and solved to extend the production of nanocomposites on large scale and their practical applications range.

Among the aforementioned nanofillers, it is worth noting that graphene enjoys a marked competitiveness, especially in terms of cost-effectiveness. It can be derived at high purity from graphite, an abundant natural resource, using relatively convenient approaches; other graphitic carbon nanofillers such as carbon nanotubes and carbon nanofibers, instead, usually require expensive and complicated equipment, as well as high energy consumption. Over time, the increased availability of graphene has meant that many researchers, already engaged in studies on polymer nanocomposites containing nanoclays or nanotubes, have increasingly turned their attention to the graphene-based ones for which dispersion issues have been successfully faced through functionalization [4,5], thermal treatments [6,7], or the use of opportune surfactants [8].

Polyamides 6 (PA6) are thermoplastic resins with interesting mechanical properties in terms of resistance and toughness such as to fully fall within the family of so-called techno polymers. Currently, they are widely used in a wide range of industrial sectors, among which the automotive one is noteworthy, where the potential offered by PA6-based materials are strongly fuelled by the continuous attempt to replace metal parts with plastic materials aimed to reduce weight and costs.

The interest of experts has so far highlighted the need to further improve the performance of PA6 compounds to meet specific and particularly demanding requirements [9,10,11], and, therefore, these materials are still the subject of intense research.

In this frame, special attention was paid to the development of new materials obtained by modifying PA6 by the inclusion of graphene-like fillers such as graphene nanoplatelets (GNPs) [12,13,14,15].

For example, Mayoral et al. [16] investigated the influence of GNPs’ content and processing conditions on the thermal, mechanical, and electrical properties of a PA6-based composite prepared by melt mixing. The results showed, among other things, that the increase in the filler content induces an improvement in the crystallinity and tensile strength of the composite. On the other hand, the increase in the rotation speed of the screws, favouring a better dispersion and distribution of the graphene agglomerates, lowers the percolation threshold and contributes to a significant increase in both the tensile modulus and the electrical conductivity of the host matrix.

Gomez et al. [17] considered polyamide-6 nanocomposites including graphene-like fillers with different surface chemistry and thickness. In detail, composite materials containing different amounts of graphene nanoplatelets (GNPs), graphene oxide (GO), reduced graphene oxide (rGO), or silane-functionalized reduced graphene oxide (f-rGO) were produced, first as a masterbatch and then diluted, by melt-extrusion. All formulations were systematically characterized in terms of morphological–structural issues, fluidity, and mechanical properties. Morphological observations by scanning electron microscopy (SEM) clearly indicated that the dilution operation, regardless of the type of filler included, drastically reduces the presence of large aggregates. In terms of processability, all the composites showed a non-monotonous trend of the melt flow index essentially due to a lubricating effect already reported in the literature for low contents of graphite particles and a subsequent increase in the viscosity of the melt as their concentration increases. Regarding the mechanical properties, flexural and tensile tests of masterbatches and diluted materials have indicated significant improvements in the modulus, tensile, and flexural ultimate strengths compared to the reference pure matrix.

Lee et al. [18] reported on PA6 composites, containing up to 17% by weight of graphene, intended for the manufacture of 3D printers’ filaments. With the aim to gain insights about the influence of the filler content on the rheological behaviour of these composites and therefore to verify the effective processability of the materials studied with conventional additive manufacturing technologies, rheological measurements in stationary and dynamic conditions have been performed on specimens with different content of graphene nanoplatelets. The rheological analysis showed that for GNP contents above the percolation threshold, there is a restriction of the Newtonian region and an intensification of the shear thinning phenomenon. Taking into account that these effects influence the 3D printability of materials, Lee et al. developed and validated a method to describe their flow through the printer nozzle.

This contribution, as part of a larger project aimed at the development of novel materials potentially usable for the production of energy storage components intended for the growing market of electrical vehicles, reports the results of a preliminary study carried out on compounds based on an injection moulding grade of PA6 and lamellar graphene, supplied by an Italian start-up. To obtain easily scalable solutions, the examination focused on samples, including various amount of the carbonaceous filler (up to 5 wt%), prepared and potentially processable with technologies traditionally used in the reference mass sector.

Melt compounded samples were transformed into plates by compression moulding from which specimens of suitable size were obtained for the subsequent static-mechanical tensile and flexural tests. The experimental evaluations revealed the effect of the GNPs content on the main mechanical parameters such as modulus and ultimate strengths and, therefore, highlighted their performances compared with the pure matrix processed under the same conditions. 

Scanning electron microscope observations of tensile-fractured surfaces of the specimens provided information about the effective dispersion level of the filler, actually achieved under the applied process conditions, supporting mechanical behaviour detected for studied materials.

## 2. Materials and Methods

### 2.1. Materials

The research was focused on compounds constituted by a polyamide 6 (PA6) resin supplied by Ravago Group under the trade name Ravamid B NC (density: 1.09 g/cm^3^, HDT: 50 °C) as the matrix and graphene nanoplatelets (GNPs) purchased as G2Nan at Nanesa S.r.l. (Arezzo, Italy) as the reinforcing phase. The latter, having a density of 2.2 mg/cm^3^, a Young’s modulus equal to 1 TPa and a tensile strength of 5 GPa, is available in the form of flakes with diameters between 10 and 20 µm and thickness between 5 and 20 nm.

### 2.2. Compound Preparation

The raw materials, pre-dried in an oven under vacuum for 3 h at a temperature of 100 °C, were extruded with the aid of a co-rotating twin screw extruder from Collin Teachline ZK25T (Ebersberg, Germany) by setting the screw speed at 40 rpm and the screw temperature profile 25–240–250–250–230–220 °C from the hopper to the die.

Two-millimetre-thick plates were prepared by compression moulding at 250 °C, using a Collin GmbH (Edersberg, Germany) model P400E press and applying the following pressure profile over time: 3 min at 0 bar, 1 min at 2 bar, and subsequent maintenance of the pressure during the cooling phase of the sample up to room temperature to prevent any shrinkage of the material, which, in this last phase, would generate, among other things, undesirable aesthetic defects of the products.

### 2.3. Characterization Techniques

The thermal analysis of the compounds and of the pure PA6 matrix, chosen as a reference, was carried out by differential scanning calorimetry using a DSC instrument from TA Instruments, model Q2000.

The tests were carried out at a scanning speed of 10 °C/min on samples of 8–10 mg, applying a typical step-by-step procedure: heating from room temperature to 250 °C, isothermal stasis at 250 °C for 1 min, cooling from 250 °C to room temperature, and subsequent heating again up to 250 °C. A thermal rate of 10 °C/min was applied in all non-isothermal stages. The mean values and standard deviations of these parameters are the result of at least three tests performed on each compound. In this analysis, as in the following ones, the compounds are compared with the pure matrix processed under the same conditions.

The processing of the thermograms was mainly limited to those relating to the second heating run to exclude any influences of the previous thermomechanical history (processing) of the material on the thermal parameters such as temperature (*T_m_*) and melting enthalpy (Δ*H*, characteristic of the same). Furthermore, the average values of the melting enthalpies were used to estimate the degree of crystallinity of the materials studied in accordance with the following simple expression:

(1) $$X_{c}\left(\%\right)=\frac{∆H_{m}}{\left(1-X_{f}\right)∆H_{m}^{0}}\cdot 100$$

where *X_c_* is the crystallinity degree, *X_f_* is the weight fraction of the filler, and $∆H_{m}^{0}$ is the enthalpy of melting of the 100% crystalline material equal to 230 J/g for PA6 [19].

Thermogravimetric measurements, on the other hand, were carried out with a Thermogravimetric Analyzer model Pyris Diamond TGA (Perkin Elmer), which allows the recording of the weight loss of the material examined as a function of temperature. In this regard, all the extruded samples were heated from 25 to 600 °C in nitrogen and from 600 to 800 °C in air, with a heating rate of 10 °C/min.

The processing of typical diagrams percentage of weight loss vs. temperature and their derivative graphs provided some characteristic data of the tested material such as temperatures corresponding to a weight loss of 5% (T_5%_) and to the maximum rate of thermal degradation (T_d_), the maximum rate of weight loss (R), and the percentage of residue at the end of the test. The repeatability of the results was verified by performing at least three measurements for each material.

Tensile and flexural properties of all investigated materials were determined according to the ASTM 3039 and the ASTM D790 Standard, respectively. All mechanical tests were accomplished using a universal dynamometer Instron 4505 equipped with a load cell of 1 kN and setting a constant crosshead speed at 2 mm/min. In more detail, for tensile measurements, dumbbell-shaped specimens with a central section 4 mm wide and 2 mm thick were loaded between 2 grips placed at a distance of 26 mm. Regarding flexural tests, instead, parallelepiped specimens (11.5 × 100 × 2 mm) were loaded according to a three-point bending configuration. In both cases, at least 5 measurements were carried out for each material to verify the repeatability of the data (modulus and strength) that will be later provided as mean values and corresponding standard deviations.

Morphological observations of sections obtained by cryogenic fracture of compression-moulded samples were collected with the aid of a field emission scanning electron microscope (SEM) (mod. FEI QUANTA 200 F) (Zurich, Switzerland) operating in high vacuum conditions at the voltage of 20 kV. The observed sections were subjected to a preliminary metallization with a gold–palladium alloy in order to make them suitably conductive.

## 3. Results and Discussion

### 3.1. Thermal Properties

**Figure 1:**
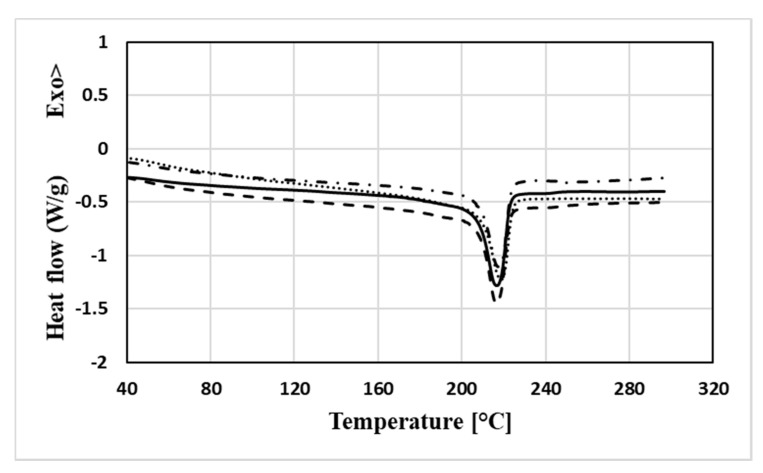
Calorimetric curves relating to the second heating ramp (solid line: PA6, dashed line: PA6 + 1% GNP, dash and dot line: PA6 + 3% GNP, dotted line: PA6 + 5% GNP).

Figure 1 compares the thermograms of the second thermal scan of the compounds examined and of the reference pure polyamide PA6, suitably shifted to allow for an improved resolution of the same. All curves present a single endothermic melting signal whose shape and position do not appear to be influenced by the content of graphene nanoplatelets. Actually, from the data collected in Table 1, obtained by processing these curves in terms of peak temperature and signal area, corresponding as already mentioned to the melting enthalpy of the material, it is clearly noted that while the signals are always centred around the temperature of 217 °C, the enthalpy parameter undergoes an almost monotonous reduction at least for increases in concentration up to 3% by weight of the carbonaceous filler. Further increases in the filler content do not seem to cause significant reductions in the same parameter, which is around 47 J/g. This trend, reflecting a decrease in the degree of crystallinity of the matrix, which goes from 24.9% for the pure PA6 matrix to 21.1% for the compound containing 3% by weight of GNPs up to the mean value of 20.5% for the compound loaded at 5% by weight, can be explained by assuming that the effective dispersion of the filler hinders the structural organization of the host matrix during the cooling of the compounds.

**Figure 2:**
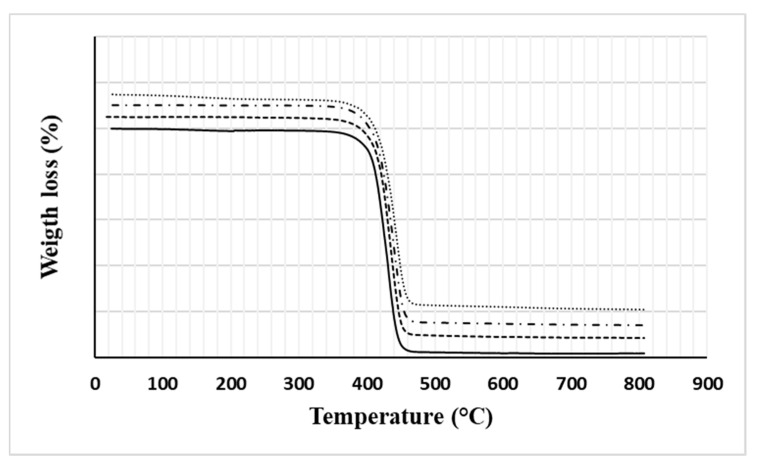
TG curves of all investigated materials (solid line: PA6, dashed line: PA6 + 1% GNP, dashed and dotted line: PA6 + 3% GNP, dotted line: PA6 + 5% GNP).

**Figure 3:**
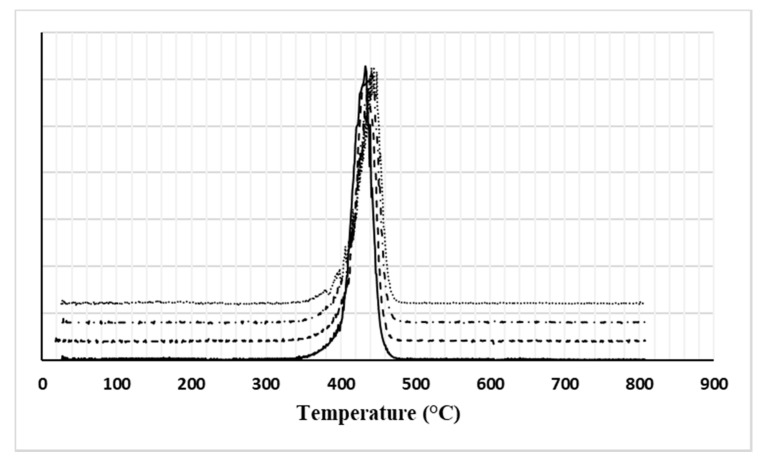
DTG traces of all investigated materials (solid line: PA6, dashed line: PA6 + 1% GNP, dashed and dotted line: PA6 + 3% GNP, dotted line: PA6 + 5% GNP).

Thermogravimetric curves of neat PA6 and its compounds are shown in Figure 2, while derivative curves are collected in Figure 3. In both cases, the curves are suitably shifted upwards, one with respect to the other, for clarity of exposure.

Given that all the materials examined show a one-step decomposition trend, from the mean values of the thermogravimetric parameters obtained from the processing of these curves and summarized in Table 2, it is clear that:

(a) The temperature corresponding to the 5% weight reduction, reasonably associated with the thermal stability of the materials, is improved by increasing the filler content. This effect is usually attributed to the consolidated ability of graphene to prevent the emission of volatiles and, thanks to its lamellar structure, to hinder the permeation of oxygen (barrier effect) [20].
(b) The inclusion of GNPs also promotes a slowing down of the degradation kinetics as the filler content increases. This effect, attributable to the organization of graphene flakes in rigid mesostructures hindering the progress of degradation phenomena, is manifested by the slight shift of the peak temperature (T_d_) towards higher values, especially for GNP contents up to 3% by weight as well as by the progressive reduction of the peak height (P_H_) compared to the pure reference PA6 matrix.
(c) The amount of the residue (ashes) at 800 °C increases from approximately 2%, for the neat matrix, up to about 6%, measured for the compound with the highest GNPs content. This trend, although it does not faithfully reflect the added filler content, reasonably shows a monotonically increasing trend. Among other things, these data also depend on the degradation mechanisms of the GNP used, governed, in turn, by the possible presence of residues of surfactants used during the industrial synthesis of the product to favour its exfoliation [21], degradation of any labile functional groups [22], and/or impurities usually not detailed in the technical data sheet of the carbonaceous filler. In this regard, it is worth pointing out that the technical data sheet of the G2Nan filler reports a residue at 800 °C, derived from a thermogravimetric analysis in nitrogen, which amounts to about 3.8% by weight but does not provide any information regarding the nature of the same.

### 3.2. Quasi-Static Mechanical Behaviour

The tensile properties of the examined compounds are compared with those of the neat PA6 in Table 3. It is evident that as the GNP content increases, Young’s modulus increases monotonously, while an opposite trend is shown about the strain at break. These results can be explained taking into account that the modulus, among others, depends on the stiffness of the filler and the crystallinity of the matrix, while the strain at break is influenced by the filler–matrix interactions, the aspect ratio of the filler, the level of dispersion actually obtained during the preparation of the specimens, and any boundary defect generated during the cutting of the specimen, and, of course, could induce premature failure of the same.

In our case, given the reduction of the degree of crystallinity of the PA6 matrix in the presence of GNPs already discussed above, it can be assumed that the increase in Young’s modulus is mainly determined by the high stiffness of the added graphene flakes. As regards the strain at break, excluding chemical interfacial interactions between the GNP, hydrophilic and non-functionalized, and the hydrophobic PA6 matrix, the reduction of this parameter, very common in many particle-filled polymers, can be reasonably attributed to a lower probability of exfoliation of the graphene lamellae with increasing their content. The segregation of the graphene flakes generates macro-defects acting as stress concentration points, which induce cracks and favour the premature failure of the tested specimen.

As regards the tensile strength, apparently unchanged at too low filler content (1% by weight), the results indicated that this parameter is increased for GNP contents up to 3 wt% but reverses its trend at the concentration of 5 wt%.

In this regard, it is well known that it is difficult to predict the trend in tensile strength, because it not only depends on variables to which the modulus is also sensitive, such as the size of the aggregates of the dispersed phase and the crystallinity of the matrix, but also is strongly influenced by the polymer–filler interaction. In our case, as the filler content increases, alongside the reduction of the crystallinity of the matrix already discussed above, it is also reasonable to expect a worsening of the dispersion of GNPs with an increase in the size of the graphene aggregates. These considerations, together with the foreseeable poor interfacial interaction, justify the reduction of the tensile strength over the concentration range 3–5 wt% [23,24].

**Figure 4:**
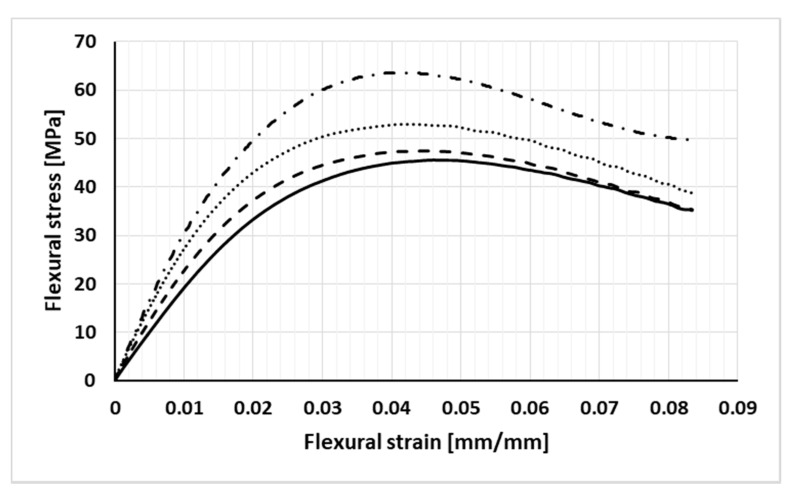
Representative stress–strain flexural curves of investigated samples (solid line: PA6, dashed line: PA6 + 1% GNP, dashed and dotted line: PA6 + 3% GNP, dotted line: PA6 + 5% GNP).

Representative flexural stress–strain curves of PA6/GNPs compounds and reference material (neat PA6) are shown in Figure 4. The elaboration of these curves has provided mean values of flexural modulus and strength collected in Table 4.

In short, the experimental tests have shown mean values of the flexural stiffness (modulus) visibly increasing with respect to the neat matrix at least up to a content of GNPs equal to 3% by weight; further increases in filler concentration do not seem to significantly affect this parameter.

The flexural strength of compounds, on the other hand, is always higher than the neat PA6 one. In particular, this parameter shows a trend similar to that described for the tensile strength: a nearly constant value at low filler content (1 wt%), an increase of about 30% for the compound at 3 wt% of GNPs, and a subsequent reduction with the further increase in the concentration of graphene up to an average value of about 54 MPa, still 18% higher than the one characterizing the pure matrix.

In flexural loading, the upper part of the specimen undergoes compressive stresses while the lower part of the same is subjected to tensile stresses. That said, for the 3%-by-weight GNP-filled system, the significant increase in both stiffness and tensile strength can justify the benefits found also in terms of flexural strength offered by the side of the specimen opposite to the one subjected to the concentrated bending load.

For the more concentrated compound, despite the further increase in tensile stiffness, the reduction of both tensile and flexural strength was attributed to a worsening of the dispersion of graphene flakes with a higher probability of inclusion of voids in the sample.

### 3.3. Morphological Analysis

**Figure 5:**
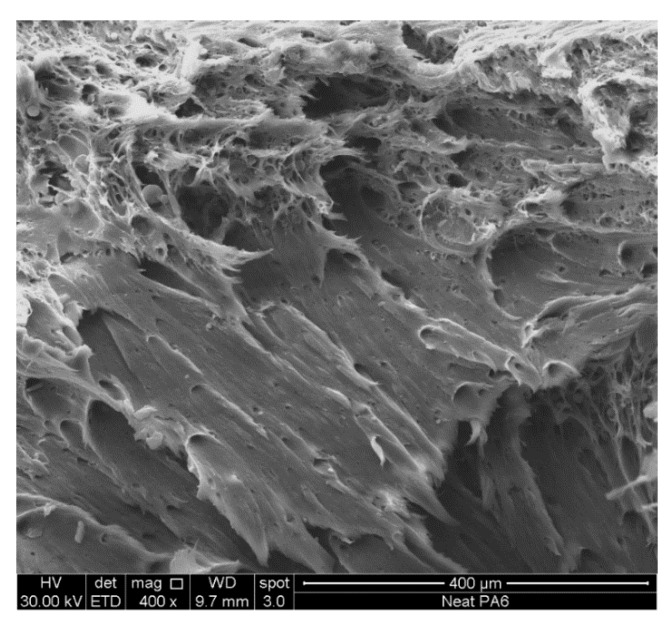
SEM micrograph of neat PA6.

**Figure 6:**
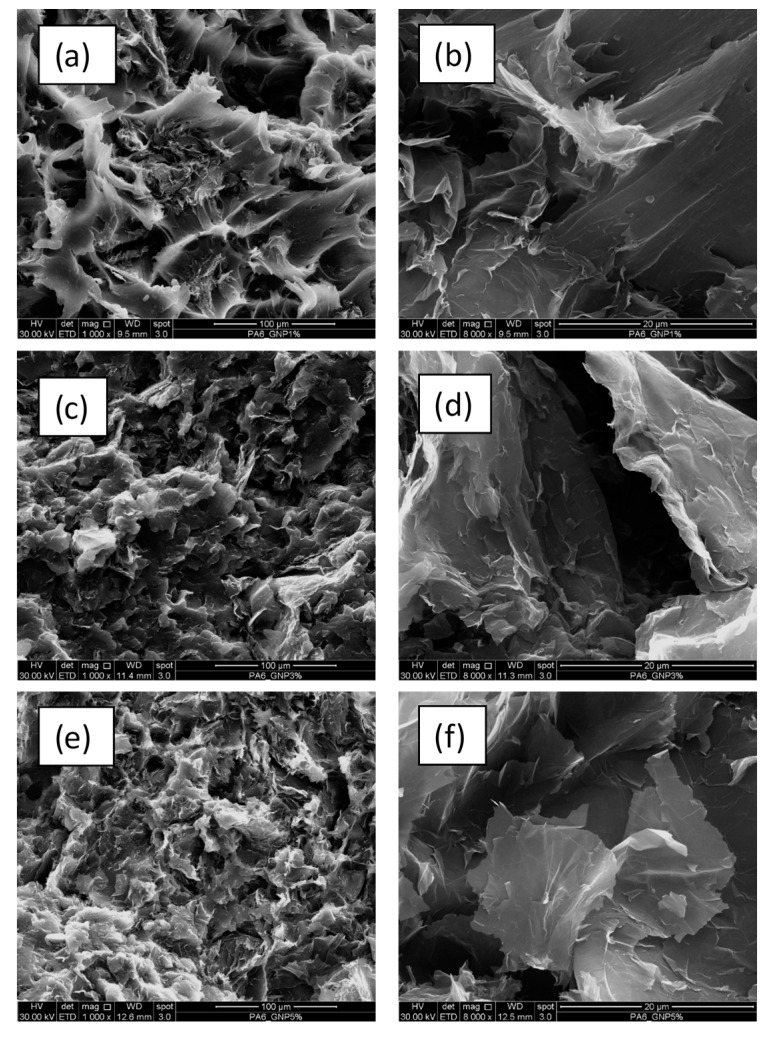
SEM micrographs collected at two different magnifications (1000× and 8000×) for PA6 compounds with different content of GNPs (**a**,**b**) 1 wt%, (**c**,**d**) 3 wt%, and (**e**,**f**) 5 wt%, respectively.

Figure 5 and Figure 6 show the SEM images of the fractured surfaces obtained with tensile tests for the PA6 neat specimens and for those of compounds with various content of graphene nanoplatelets.

The microscopic observation of neat PA6 (Figure 5) shows the occurrence of yield phenomena with evident plastic deformations generated by the applied tensile stress, confirming the ductile character of the matrix.

Representative SEM micrographs of the tensile fracture surfaces of the compounds (see Figure 6), on the other hand, highlights an almost uniform distribution of GNP with lamellae that surround the yield bands of the matrix and overlap more and more especially in the system filled with 5% by weight of nanoplatelets. Thus, although it can be assumed that, overall, the melt-compounding conditions used are such as to guarantee an efficient exfoliation of the GNP lamellae, for the more concentrated system examined, also given the expected increase in melt viscosity [25], the formation of aggregates that supports the compromise of mechanical parameters already discussed cannot be excluded.

## 4. Conclusions

In summary, formulations based on a polyamide 6 matrix and containing 1, 3, and 5 weight percent of a commercial graphene nanoplatelets have been prepared by melt mixing and characterized in terms of thermal and mechanical properties as well as through morphological observations of the tensile fracture surfaces.

In short, the main results obtained so far show that:

1. The inclusion of GNP hinders the crystallization of the polyamide matrix, which shows an evident reduction in the degree of crystallinity, especially for contents up to 3% by weight of filler. Further additions of GNP seem to have no major repercussions on this parameter, which, however, does not reverse this trend.
2. Graphene nanoplatelet compounds, in addition to confirming the improvement in thermal stability widely consolidated in the literature, slightly increase the maximum temperature of the derived signal and progressively reduce its height without changing its shape.
3. Tensile and bending stiffness of compounds increase with the GNP content, but the strength parameter, for both types of loading, shows a non-monotonous trend. Specifically, a non-monotonous trend was detected for this parameter essentially dominated by the inevitable aggregation phenomena of graphene sheets for contents above a threshold value usually dependent on a multitude of factors such as aspect ratio of the filler, matrix viscosity, interfacial issues, and so on. This behaviour, also favoured by the foreseeable increase in viscosity of the host molten matrix, was supported by SEM micrographs of sections of specimens broken under tensile tests.

## Figures and Tables

**Table 1:** Calorimetric parameters.

Sample	*T_m_* (°C)	Δ*H_m_* (J/g)	*X_c_* (%)
PA6	217 ± 1	57.2 ± 2.3	24.9
PA6 + 1 wt% GNPs	217 ± 3	53.7 ± 1.5	23.3
PA6 + 3 wt% GNPs	218 ± 2	48.5 ± 3.0	21.1
PA6 + 5 wt% GNPs	218 ± 1	47.1 ± 1.1	20.5

**Table 2:** Thermogravimetric data.

Sample	T_5%_ (°C)	T_d_ (°C)	P_H_ (wt%/min)	Residue at 800 °C (%)
PA6	386.0 *±* 1.5	434.0 *±* 0.8	30.7 *±* 1.7	1.7
PA6 + 1% GNP	389.1 *±* 0.8	438.9 *±* 0.5	28.3 *±* 0.8	3.5
PA6 + 3% GNP	390.2 *±* 0.9	440.7 *±* 0.4	26.6 *±* 0.8	4.1
PA6 + 5% GNP	395.3 *±* 0.5	441.1 *±* 0.6	25.3 *±* 0.6	5.8

**Table 3:** Tensile parameters.

Sample	Young’s Modulus (MPa)	Tensile Strength (MPa)	Strain at Break (%)
PA6	1041 *±* 141	44.18 *±* 0.96	29.87 *±* 1.47
PA6 + 1% GNP	1122 *±* 100	45.37 *±* 1.57	23.62 *±* 5.22
PA6 + 3% GNP	1167 *±* 113	48.11 *±* 0.69	8.64 *±* 1.33
PA6 + 5% GNP	1179 *±* 44	31.06 *±* 1.73	7.14 *±* 1.21

**Table 4:** Flexural parameters.

Sample	Modulus (MPa)	Flexural Strength (MPa)
PA6	1996 *±* 154	45.91 *±* 0.53
PA6 + 1% GNP	2405 *±* 94	46.32 *±* 2.84
PA6 + 3% GNP	3482 *±* 101	61.04 *±* 2.05
PA6 + 5% GNP	3303 *±* 198	54.15 *±* 2.06
